# Liver transplant bile duct suture leading to acute cholangitis: endoscopic removal via cholangioscopy

**DOI:** 10.1055/a-2285-2546

**Published:** 2024-04-09

**Authors:** Emil Thyssen, Parsia Vagefi, Arjmand Mufti, Thomas Tielleman

**Affiliations:** 1 12334 Department of Internal Medicine – Digestive and Liver Diseases, The University of Texas Southwestern Medical Center, Dallas, United States; 2 Department of Surgery – Transplant Surgery, The University of Texas Southwestern Medical Center, Dallas, United States

Acute cholangitis occurs from biliary obstruction. Gallstones, strictures and neoplasia account for most cases 1 . Sutures from prior surgical interventions have previously been described as leading to obstruction 2 . We present a case of a patient who had undergone orthotopic liver transplantation with duct-to-duct anastomosis 4 years previously and developed acute cholangitis ultimately due to a retained suture at the site of the biliary anastomosis ( Video 1 ). This suture was successfully removed endoscopically to prevent future recurrences.

Endoscopic removal of liver transplant bile duct anastomotic suture via cholangioscopy forceps.Video 1

A 52-year-old woman with a history of cirrhosis secondary to primary biliary cholangitis, status post orthotopic liver transplantation (with duct-to-duct biliary anastomosis), presented 4 years after transplantation with acute right upper quadrant abdominal pain, nausea, and vomiting. She was found to have obstructive jaundice and sepsis consistent with acute cholangitis.

Initial computed tomography (CT) revealed intrahepatic biliary dilation and a 2.7-cm filling defect in the common bile duct ( Fig. 1 ). She underwent endoscopic retrograde cholangiopancreatography (ERCP) which revealed pus emerging from the major papilla and a large stone at the middle third of the common bile duct ( Fig. 2 ). The stone was too large for removal through a nondilated distal common bile duct. Electrohydraulic lithotripsy (EHL) was not performed, given the active cholangitis. Two transpapillary plastic stents were placed for source control.

**Fig. 1:**
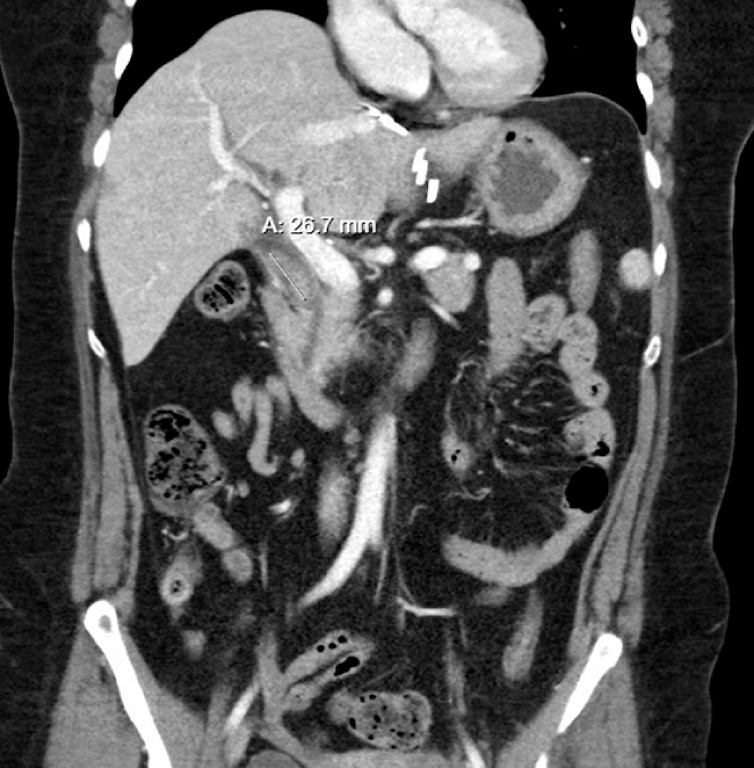
Initial computed tomography demonstrating 2.7-mm defect in the common bile duct.

**Fig. 2:**
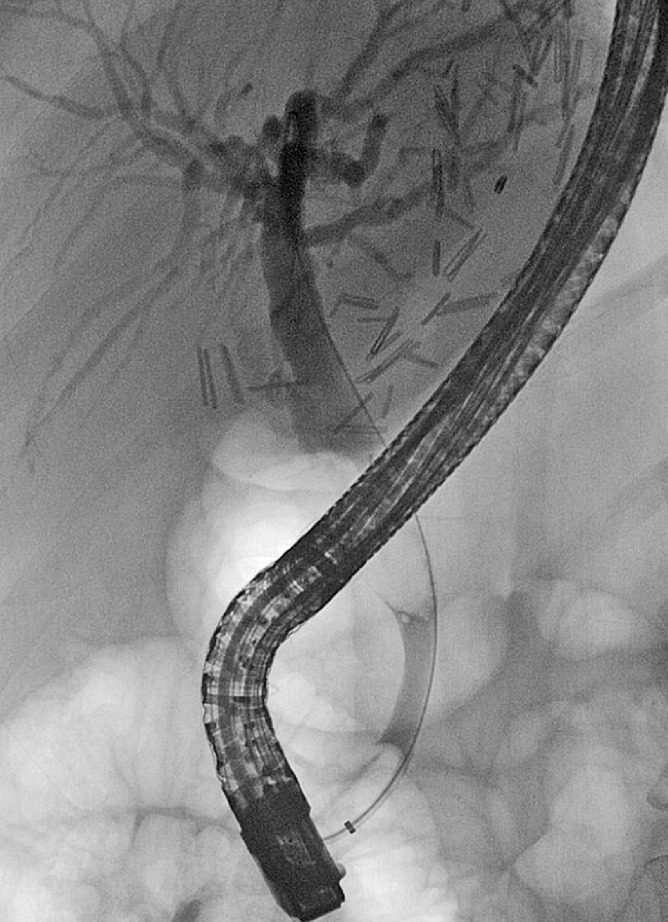
Cholangiography demonstrating a large, common bile duct stone.

ERCP was repeated 4 weeks later for stone removal. Cholangioscopy was performed, at which time EHL was successful and revealed that the stone had formed around an anastomotic suture ( Fig. 3 ). A cholangioscopy forceps was utilized for suture removal ( Fig. 4 ). The final cholangiogram ( Fig. 5 ) revealed no remaining obstruction or bile duct injury.

**Fig. 3:**
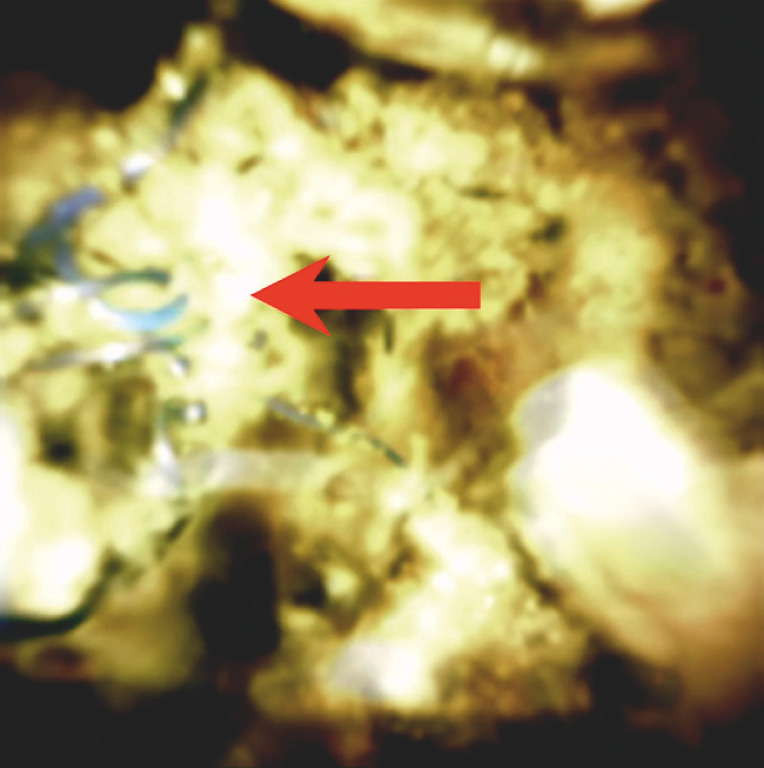
Biliary suture present and serving as a nidus to obstruction.

**Fig. 4:**
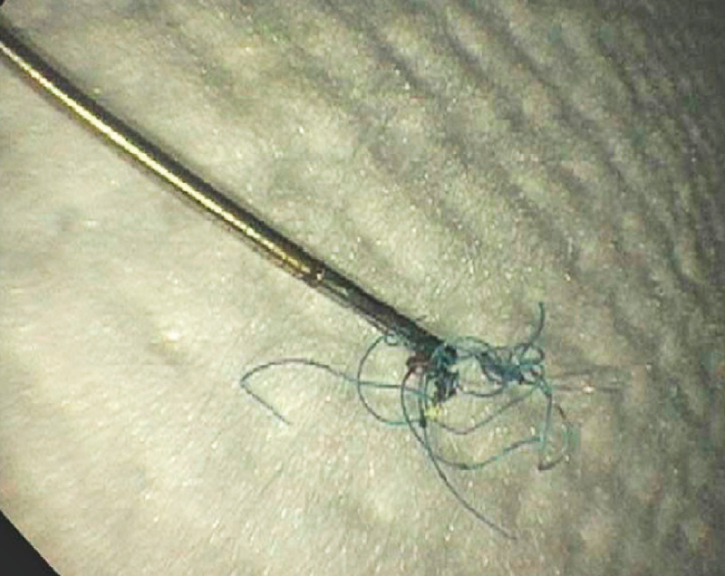
Endoscopically removed bile duct suture.

**Fig. 5:**
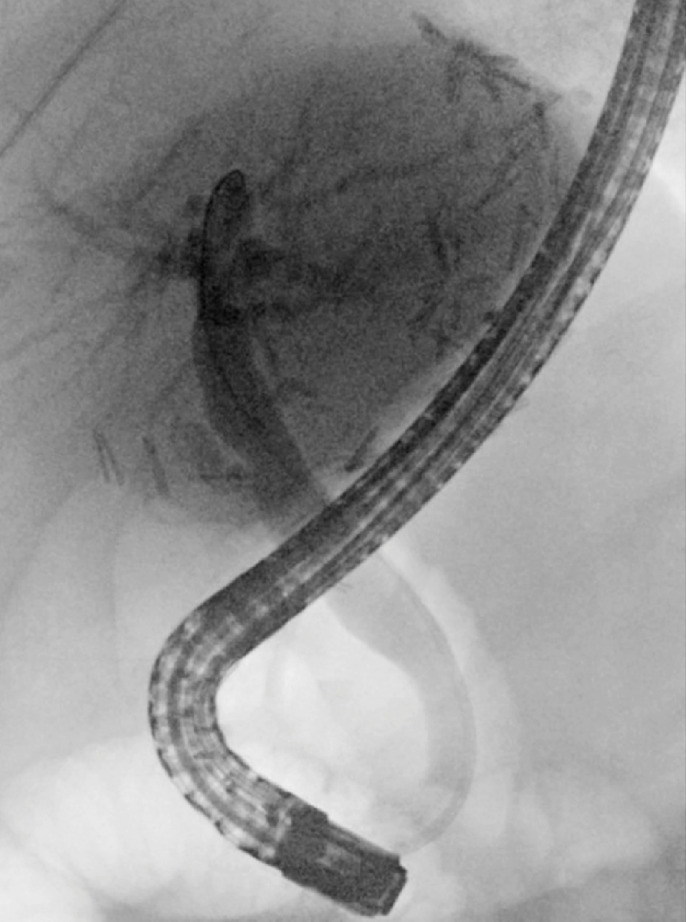
Cholangiography demonstrating resolution of obstruction after endoscopic removal of stone and suture.

This case demonstrates unique features including a prior biliary duct-to-duct anastomotic suture acting as a nidus for stone formation. Such sutures should be removed to prevent recurrent stone formation and we demonstrate that this can be safely performed endoscopically.

Endoscopy_UCTN_Code_TTT_1AR_2AG

## Notes

Endoscopy E-Videos https://eref.thieme.de/e-videos
*E-Videos* is an open access online section of the journal *Endoscopy* , reporting on interesting cases and new techniques in gastroenterological endoscopy. All papers include a high-quality video and are published with a Creative Commons CC-BY license. Endoscopy E-Videos qualify for HINARI discounts and waivers and eligibility is automatically checked during the submission process. We grant 100% waivers to articles whose corresponding authors are based in Group A countries and 50% waivers to those who are based in Group B countries as classified by Research4Life (see: https://www.research4life.org/access/eligibility/ ). This section has its own submission website at https://mc.manuscriptcentral.com/e-videos .
